# Spatio-temporal dynamics of rabies and habitat suitability of the common marmoset *Callithrix jacchus* in Brazil

**DOI:** 10.1371/journal.pntd.0010254

**Published:** 2022-03-31

**Authors:** Julio A. Benavides, Ram K. Raghavan, Vanner Boere, Silene Rocha, Marcelo Y. Wada, Alexander Vargas, Fernanda Voietta, Ita de Oliveira e Silva, Silvana Leal, Alene de Castro, Maria de Fatima Arruda, A. Townsend Peterson, Jane Megid, Maria Luiza Carrieri, Ivanete Kotait

**Affiliations:** 1 Doctorado en Medicina de la Conservación y Centro de Investigación para la Sustentabilidad, Facultad de Ciencias de la Vida, Universidad Andres Bello, República 440 Santiago, Chile; 2 MIVEGEC, IRD, CNRS, Université de Montpellier, Montpellier, France; 3 Departamento Higiene Veterinária e Saúde Pública, Faculdade de Medicina Veterinária e Zootecnia, Universidade Estadual Júlio de Mesquita Filho, Botucatu, Brazil; 4 Department of Veterinary Pathobiology, College of Veterinary Medicine, University of Missouri, Columbia, Missouri, United States of America; 5 Department of Public Health, School of Health Professions, University of Missouri, Columbia, Missouri, United States of America; 6 Institute of Humanities, Arts and Sciences, Federal University of Southern Bahia -UFSB, Itabuna, Brazil; 7 Secretaria de Vigilância em Saúde (SVS), Ministério da Saúde, Brasilia, Brazil; 8 Secretaria de saúde de Pernambuco, Recife, Brazil; 9 Programa Estadual de Vigilância de Epizootia, Secretaria de saúde de Rio Grande do Norte, Natal, Brazil; 10 Setor de Psicobiologia, Departamento de Fisiología Universidade Federal do Río Grande do Norte, Natal, Brazil; 11 Biodiversity Institute, University of Kansas, Lawrence, Kansas, United States of America; 12 Instituto Butantan, São Paulo, Brazil; 13 Retired Researcher, Instituto Biológico, São Paulo, Brazil; Universitetet i Oslo, NORWAY

## Abstract

Rabies transmitted by wildlife is now the main source of human rabies in the Americas. The common marmoset, *Callithrix jacchus*, is considered a reservoir of rabies causing sporadic and unpredictable human deaths in Brazil, but the extent of the spillover risk to humans remains unknown. In this study, we described the spatiotemporal dynamics of rabies affecting *C*. *jacchus* reported to Brazil’s Ministry of Health passive surveillance system between 2008 and 2020, and combined ecological niche modelling with *C*. *jacchus* occurrence data to predict its suitable habitat. Our results show that 67 outbreaks (91 cases) of rabies affecting *C*. *jacchus* were reported by 41 municipalities between January 2008 and October 2020, with a mean of 5 outbreaks/year [range: 1–14]. The maximum number of outbreaks and municipalities reporting cases occurred in 2018, coinciding with higher surveillance of primate deaths due to Yellow Fever. A mean of 3 [1–9] new municipalities reported outbreaks yearly, suggesting potential spatial expansions of the *C*. *jacchus* variant in northeastern Brazil and emerging rabies spillover from vampire bat *Desmodus rotundus* to *C*. *jacchus* in the north and south. Outbreaks were concentrated in the states of Ceará (72%) and Pernambuco (16%) up to 2012, but are now reported in Piauí since 2013, in Bahia since 2017 (*D*. *rotundus’* antigenic variant, AgV3) and in Rio de Janeiro since 2019 (AgV3). Besides confirming suitable habitat for this primate in the northeast and the east coast of Brazil, our Maximum Entropy model also predicted suitable habitat on the north and the west states of the country but predicted low habitat suitability among inland municipalities of the Caatinga biome reporting rabies. Our findings revealed new areas reporting rabies infecting *C*. *jacchus*, highlighting the need to implement strategies limiting spillover to humans and to better understand the drivers of *C*. *jacchus* rabies dynamics.

## Author summary

Rabies virus is the deadliest virus affecting mammals. In Brazil, rabies transmitted by the common marmoset primate is emerging and causing unpredictable human deaths. This primate once endemic to the northeast of the country has now invaded regions in the south throughout human-mediated introductions. However, the dynamics of rabies in this primate and the extend of spillover risk to humans remain unknown. We found that outbreaks of rabies in this marmoset reported to the Ministry of Health are continuously reported in new areas, including three new states since 2012 and three states reporting rabies originated from the common vampire bat. We also showed that this primate has suitable habitat to invade other states in the north and west of Brazil. Preventive strategies should be implemented to limit further rabies spillover to humans in new areas.

## Introduction

Rabies virus (RABV) causes the deadliest disease among mammals and is directly transmitted through the bite of infectious animals [1]. In the Americas, rabies transmitted by wild animals is now the main source of human deaths associated with rabies [2]. Most prevention and control efforts targeting rabies transmitted by wildlife have focused on bats such as the common vampire bat *Desmodus rotundus*, which is responsible for the majority of human rabies deaths in the continent [3]. However, rabies is also emerging in other wild mammals including primates and foxes hosting specific RABV variants that have caused sporadic human deaths during unpredictable spillover events [4–6]. Despite common interactions between wild primates and humans [7,8] and thousands of humans bitten every year by primates that could expose them to diseases including rabies in tropical countries such as Brazil [9], our knowledge on the distribution and epidemiology of rabies transmitted by primates remains poorly understood, limiting the implementation of preventive measures to avoid rabies spillover to humans.

The common marmoset, *Callithrix jacchus*, is a New World primate endemic to northeastern Brazil [10]. Rabies in the common marmoset and humans bitten by this primate have been sporadically detected in Brazil [6,11–13], resulting in the report of nine human deaths associated with rabies transmitted by *C*. *jacchus* between 2000 and 2017 [6]. The common marmoset is considered the only primate reservoir of rabies, with genetic characterization of the viral strain showing at least one specific rabies variant circulating among the common marmoset [4]. However, the extent of the rabies risk posed by this primate remains unknown. In the wild, common marmosets are found in scrub, forest, and plantation habitats in the Atlantic coastal forest as well as the semi-deciduous Caatinga biome [10,14,15]. However, human-mediated transportation and pet trade have introduced the common marmoset into both urban areas and southern regions of the country including the states of Rio de Janeiro and Minas Gerais [13,16]. This expansion of the *C*. *jacchus* distribution can result in increasing interactions between this species and humans, which could lead to an increase in bites to humans and subsequent risk of rabies spillover.

To evaluate the spillover risk posed by common marmosets to humans, it is crucial to understand the spatial distribution of this primate and the circulation of rabies affecting this species. Ecological niche modelling can identify suitable habitat for the further human-mediated invasion of *C*. *jacchus*, which would help anticipate future hotspots of rabies spillover risk as previously done for other wild reservoirs such as the common vampire bat [17]. Despite being one of the most studied primates in Brazil, there are only anecdotal reports of its occurrence, related to long-term studied groups by primatologists [18]. To fulfill the incomplete information on a species distribution, the potential geographic distribution and habitat suitability can be reliably determined by presence-only occurrence data and environmental data using ecological niche modeling approaches [19,20]. In particular, the maximum entropy approach has been widely used to model spatial distribution of species, in which presence-only occurrence data is combined with environmental data to produce statistically robust predictions of geographic suitability for a species [21]. Two recent studies have used this approach to predict the potential areas of hybridization between the common marmoset and other marmoset species on their native range [22] and the east of the country [23] using published data on marmoset occurrences.

The aim of this study was to describe the spatiotemporal patterns of rabies cases in *C*. *jacchus* reported by state health services to Brazil’s Ministry of health passive surveillance system between 2008 and 2020, and to identify whether spatial expansions of rabies are occurring in this species. We also combined published and newly collected occurrence data of the common marmoset to estimate its current range and suitable habitat distribution across Brazil, including recent methodological improvements to ecological niche modeling to identify the environmental drivers influencing *C*. *jacchus* distribution.

## Materials and methods

### Datasets

#### (i) Reports of rabies infections in *C*. *jacchus* to the Brazilian Ministry of Health from 2008 to 2020

Dead *C*. *jacchus* suspected to have died from rabies were passively reported by state’s health units to the Rabies Technical Group of the Ministry of Health’s Zoonoses Surveillance Technical Unit. This unit received post mortem brain samples and performed laboratory confirmation using the fluorescent antibody test (FAT) by partnering with reference laboratories, and performed antigenic characterization to determine rabies variant typing on a subset (39%) of positive samples including at least one sample per outbreak [4]. We analyzed data as either municipality-level outbreaks (defined as at least one laboratory-confirmed case of rabies submitted by a municipality within a year) or cases (defined as the total number of laboratory-confirmed cases submitted by a municipality within a year). To evaluate if temporal trends in rabies-positive *C*. *jacchus* were influenced by passive surveillance effort, we also included the total number of dead non-human primates reported to the Ministry of Health’s Zoonoses Surveillance Technical Unit, which includes mainly primates tested for Yellow Fever and rabies. Although this data was not detailed by primate species, it can represent a proxy of passive surveillance effort at the national and state level. The dataset is available on S1 and S2 Tables of the Supplementary Material.

#### (ii) *C*. *jacchus* occurrence data

A total of 178 GPS locations of *C*. *jacchus* were obtained from four different data sources: (i) by direct observation of the species during a field mission in three states in 2019 (number of occurrences N = 21), (ii) from published GPS occurrence data extracted from the ATLANTIC PRIMATES database (N = 117) recorded from 1987 to 2017 [18], (iii) by requesting GPS locations of two wild *C*. *jacchus* groups studied for more than a decade in the state of Rio Grande do Norte (MFA, pers. comm) (N = 2) recorded in 2019 and (iv) extracting GPS locations from recent published data available in Moraes *et al*. 2019 (excluding locations already present in the ATLANTIC PRIMATES database) (N = 38) recorded from 1999 to 2017. GPS locations of *C*. *jacchus* were recorded in the field during 10-day field excursions in the states of Bahia (number of occurrences N = 8), Pernambuco (N = 10) and Rio Grande do Norte (N = 3) during January and February 2019. During these excursions, a team including at least two observers with a minimum of one experienced primatologist visually confirmed the presence of *C*. *jacchus* from a list of places that were previously suggested as habitat of *C*. *jacchus* by either the State Health Secretary that monitors this species or by primatologists working in each state. Identification of *C*. *jacchus* was performed using species-specific characteristics including their distinctive white tufted-ears and vocalizations. Data on locations is available on S3 Table. Occurrence data did not include dead animals from passive surveillance, as GPS locations for these animals were not available.

#### (iii) Data used in the Ecological niche modelling

GPS locations were first converted to an ESRI point shapefile with a defined projection of WGS 1984 and subsequently projected into WGS 1984 World Equidistant Conic projected coordinate system in ArcGIS. The presence of spatial autocorrelation in occurrence datasets is a known problem in niche modeling, whose use without rectification can result in biased estimates of distributions and generate model overfitting [24]. Therefore, we rarefied the occurrence dataset used in our ecological niche model by randomly removing locations that were within a 10, 25 or 50 km radius using the SDM Toolbox v2.2 [24,25], and selected the most appropriate radius based on visual inspection of occurrence clustering (S1 Text). The rarefied occurrence points were then randomly split in half into two groups using a random number generator in Microsoft Excel, and each group was used for calibrating and testing the niche models. Model calibration can be improved by defining an accessible area (M), the area to which the species has access via dispersal, reducing the effects of assumptions regarding the absence of species from areas that are not accessible [26]. No available estimate of the M area exists for *C*. *jacchus*. We therefore assumed an M area of 250km based on field observations and previous work on biomes identified as suitable for this species [22,23].

Previous studies have shown that the geographical distribution of marmoset primates including *C*. *jacchus* can be influenced by bioclimatic variables [23,27]. Therefore, similarly to Moraes *et al*. 2019, we used the 30 arc second (∼1 km) resolution CHELSA (Climatologies for the Earth’s Land Surface Areas) [28] data to model the ecological niche of *C*. *jacchus* in Brazil. The choice of this spatial resolution was made with a consideration of the spatial resolution in which the common marmoset data was gathered, also at 1km. The CHELSA data consists of downscaled model output temperature and precipitation estimates at a horizontal resolution of 30 arc second. Downscaling algorithm for temperature is mainly based on statistical downscaling of atmospheric temperatures, and the algorithm for downscaling precipitation incorporates orographic predictors including wind fields, valley exposition, and boundary layer height, with a subsequent bias correction [28]. The resulting data consist of a monthly temperature and precipitation data and various derived bioclimatic parameters. The 19 bioclimatic variables are specifically developed for species distribution modeling and for related ecological applications, with values for each grid or pixel representing different bioclimatic conditions of annual averages, seasonality, and extreme or limiting environmental factors for a given species. Detailed technical description of CHELSA data can be found in the technical document here: http://chelsa-climate.org/wp-admin/download-page/CHELSA_tech_specification.pdf.

### Spatiotemporal patterns in the distribution of *C*. *jacchus* rabies

Spatial dynamics of rabies outbreaks/cases in *C*. *jacchus* were explored by estimating the annual number of outbreaks/cases from January 2008 to October 2020, the annual number of municipalities reporting outbreaks and the annual number of new municipalities reporting outbreaks for the first time. We also tested the correlation between year and the number of outbreaks/cases or municipalities reporting cases using a Spearman’s correlation test in R 3.6.1 [29], since the number of outbreaks/cases was not normally distributed. For this correlation analysis, we excluded the year 2020 given incomplete data. Municipality and state maps were obtained from the open access GADM database (gadm.org), using the *getdata* and *readOGR* functions of R. All analyses were performed in R.

### Ecological niche modelling

The present climatic suitability of *C*. *jacchus* in Brazil was determined with the rarefied, presence-only occurrence data and CHELSA bioclimatic layers following the maximum entropy modeling approach previously described [30–32], with Maxent 3.4.1 software [32]. The relevance of different environmental layers was tested using the jackknife procedure in Maxent, allowing us to determine a smaller subset of variables needed to adequately describe the environmental niche for this species. First, we built a global model including 15 out of the 19 bioclimatic variables excluding variable 8 (mean temperature of the wettest quarter), 9 (mean temperature of the driest quarter), 18 (precipitation of the warmest quarter), and 19 (precipitation of coldest quarter) as they are known to have spatial artifacts that influence model outcomes [33]. Jackknife procedure allows to assess the contribution of a given variable with and without its presence to the full model. One or more least contributing variables were removed, and models refit in sequential steps, and variables retained in the last three jackknife steps were kept as three individual sets of environmental variables for model calibration (Table 1).

**Table 1:** Bioclimatic variables selected to predict *Callithrix jacchus* suitable area. Steps in the jackknife procedure to select best contributing bioclimatic variables for *Callithrix jacchus* spatial distribution in Brazil. Bioclimatic variables from sets 1, 2 and 2 were used for model calibration.

Jackknife step	Bioclimatic variables	Bioclimatic variables removed
I	All except 8, 9, 18, 19	3, 16, 13
II	Set 3: Annual Mean Temperature (1), Mean Diurnal Range (2), Temperature Seasonality (4), Max Temperature of Warmest Month (5), Min Temperature of Coldest Month (6), Temperature Annual Range (7), Mean Temperature of Warmest Quarter (10), Mean Temperature of Coldest Quarter (11), Annual Precipitation (12), Precipitation of Driest Month (14), Precipitation Seasonality (15), Precipitation of Driest Quarter (17)	4, 5, 6, 12
III	Set 2: Annual Mean Temperature (1), Mean Diurnal Range (2), Temperature Annual Range (7), Mean Temperature of Warmest Quarter (10), Mean Temperature of Coldest Quarter (11), Precipitation of Driest Month (14), Precipitation Seasonality (15), Precipitation of Driest Quarter (17)	1, 10, 14
IV	Set 1: Mean Diurnal Range (2), Temperature Annual Range (7), Mean Temperature of Coldest Quarter (11), Precipitation Seasonality (15), Precipitation of Driest Quarter (17)	none

The relationships between bioclimatic variables and (i) model response types, (ii) the type of mathematical function applied in the model [34] and (iii) the regularization multiplier (which determines how closely the model fits the observations in the environmental space) are complex and include linear, product, quadratic, threshold, and hinge correlations. Thus, exploration for optimal model performance is recommended during model calibration [35]. Therefore, we built several progressively complex models by changing the environmental variable set, the input values for regularization multiplier and the response type. The final model(s) were then selected aiming for a statistically significant parsimonious model with a low-omission rate based on three statistical criteria including the ROC criteria, omission rates and AIC values. We first ranked all models based on their significance using a partial ROC criterion (p > 0.05) and excluded all non-significant models. We then ranked the selected significant models based on the omissions rate, excluding any model with a 10% or higher omission rate. Finally, we ranked the remaining models based on their AICc values. The selected final model was replicated 10 times using the bootstrap function, and the median output was used to generate suitability maps. Model calibration and other analyses were performed using the *kuenm* package in R [36].

### Estimating *C*. *jacchus* habitat suitability in municipalities reporting rabies cases

To explore the correlation between the predicted habitat suitability of *C*. *jacchus* from our niche model and areas of rabies risk, we estimated the average habitat suitability probability derived from the model predictions for each municipality that had reported rabies cases in *C*. *jacchus*. Average probabilities per municipality polygon were calculated from a raster map of predicted habitat suitability probabilities using the *extract* function in R.

## Results

### Spatiotemporal patterns of rabies outbreaks in *C*. *jacchus*

**Fig 1:**
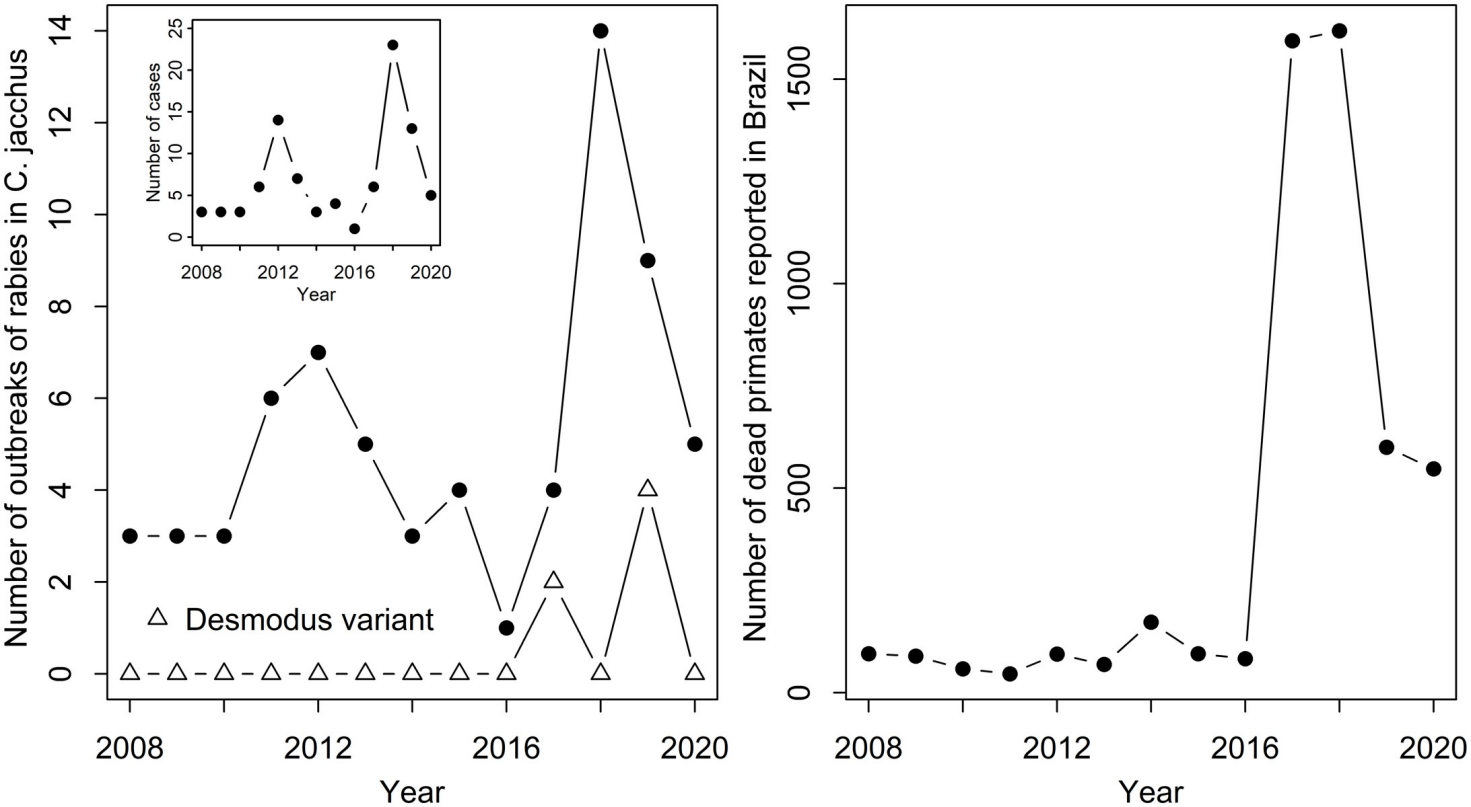
Number of outbreaks of rabies in *Callithrix jacchus* and overall dead non-human primate reported to the Brazilian Ministry of Health between 2008 and 2020. (Left) Total number of rabies outbreaks in *C*. *jacchus*. Open triangles show outbreaks belonging to the *Desmodus rotundus* variant (AgV3). The inset figure shows the total number of cases, defined as the total positive individuals reported within an outbreak. (Right) Total number of dead non-human primates reported to the Ministry of Health. Data of 2020 includes January to October.

A total of 67 outbreaks of rabies in *C*. *jacchus* comprising 91 cases (1.4 cases/outbreak [range: 1–5]) were reported to the Brazilian Ministry of health between January 2008 and October 2020. The number of outbreaks fluctuated across years with a mean of 5 outbreaks/year [1–14] and peaked in 2012 with 7 outbreaks and in 2018 with 14 outbreaks (Fig 1A). There was no significant correlation between the number of outbreaks and year (Spearman’s test, rho = 0.45, p = 0.15) nor the number of cases and year (Spearman’s test, rho = 0.44, p = 0.15) between 2008 and 2019. At the country level, there was also no significant correlation between the number of rabies cases in *C*. *jacchus* and the number of dead non-human primates reported to the Ministry of Health (Spearman’s test, rho = 0.33, p = 0.29, Fig 1B). However, the peak of rabies cases in 2018 (N = 23) coincided with a peak in the number of dead primates reported across the country (N = 1594) during an outbreak of Yellow Fever (Fig 1).

**Fig 2:**
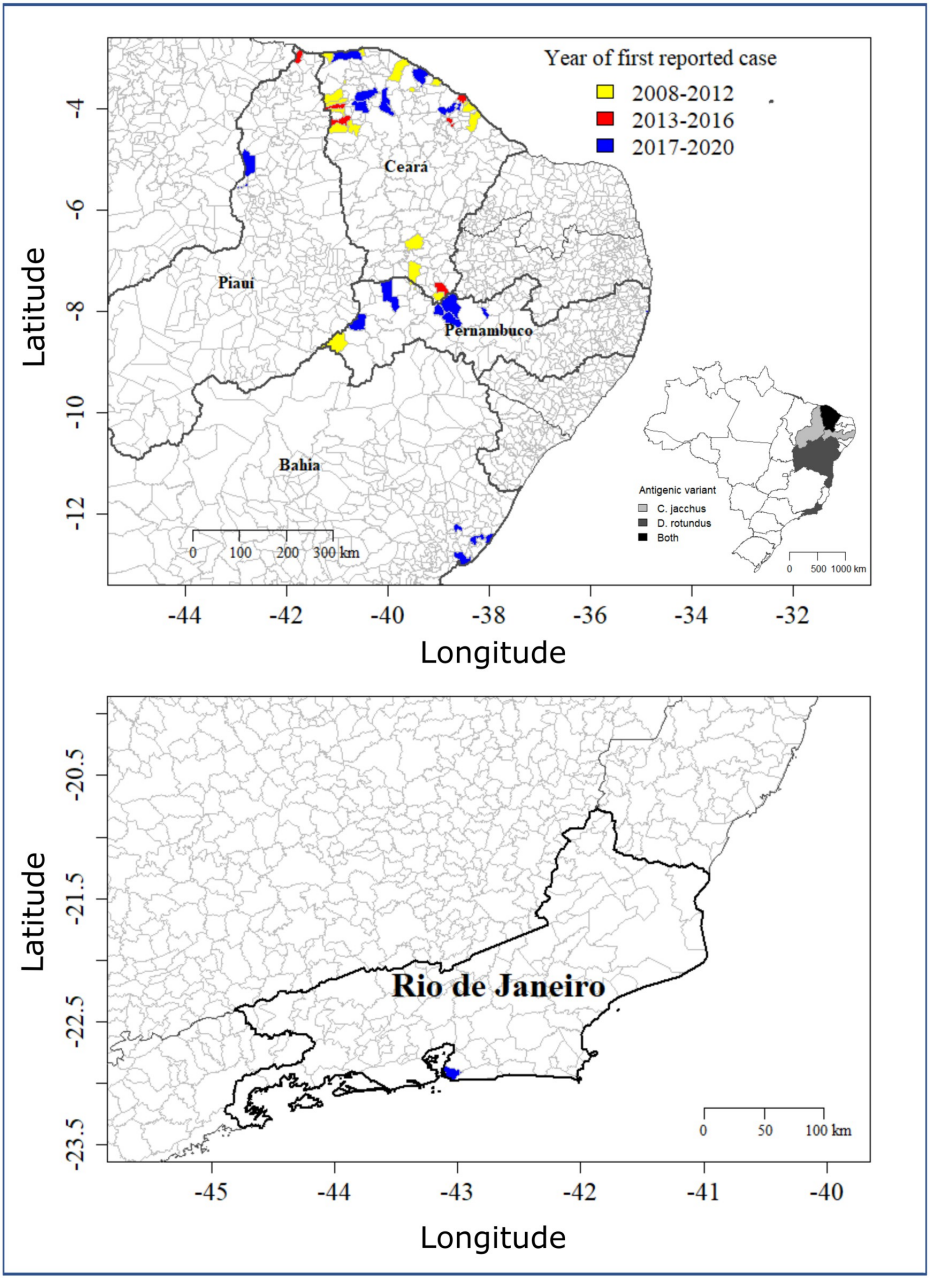
Spatial distribution of outbreaks of rabies in *Callithrix jacchus* in Brazil. (Top) The main figure shows municipalities of northeast states that have reported an outbreak since 2008, with colors representing the interval of time when the first outbreak was reported at each municipality since 2008. Inset figure shows states reporting cases, with colors representing whether states have reported rabies cases in *C*. *jacchus* belonging to the specific antigenic *C*. *jacchus* variant, the *D*. *rotundus* variant (AgV3) or both. (Bottom) Municipality location of the rabies case reported in the state of Rio de Janeiro belonging to the *D*. *rotundus* variant. Country, region and district maps were obtained from the GADM (http://www.gadm.org//) database, freely-available for academic use, using the *getData* function from the *raster* package of R (map layer can be found here: https://biogeo.ucdavis.edu/data/gadm3.6/Rsp/gadm36_BRA_2_sp.rds).

From the 67 outbreaks reported, 20 (30%) belonged to the *C*. *jacchus* antigenic variant, 41 outbreaks (61%) had no antigenic variant identified and 6 outbreaks (9%) belonged to the *Desmodus rotundus*’ antigenic variant (AgV3). Variant identification started in 2017. Outbreaks were reported in five states including the northeast states of Ceará (48 out of 67 outbreaks, 72%), Pernambuco (11, 16%), Bahia (4, 6%), Piauí (3, 4%) and the southern state of Rio de Janeiro (1, 2%) (Fig 2). All outbreaks reported in Bahia and Rio de Janeiro belonged to the *D*. *rotundus*’ antigenic variant, whereas one outbreak in *C*. *jacchus* from Ceará was also confirmed as belonging to the *D*. *rotundus*’ antigenic variant in 2019. In the state of Ceará, the annual number of rabies cases in *C*. *jacchus* reported was significantly correlated to the annual number of dead non-human primates reported to the Ministry of Health (Spearman’s test, rho = 0.69, p = 0.01). However, there was no such correlation in any other state. The total number of non-human primate deaths reported by each state varied from 0 to 1376 (median, mean±SD: 45, 192 ±381 deaths/state) between 2008 and 2020. The five states reporting cases of rabies in *C*. *jacchus* reported more non-human primate deaths between 2008 and 2020 (median, mean±SD: 172, 278 ±390 deaths/state) than the other 22 states not reporting rabies (35, 172 ±386 deaths/state). However, the state of Ceará reported fewer dead primates (n = 172) than the national average per state and Piauí reported only 12 dead primates.

**Fig 3:**
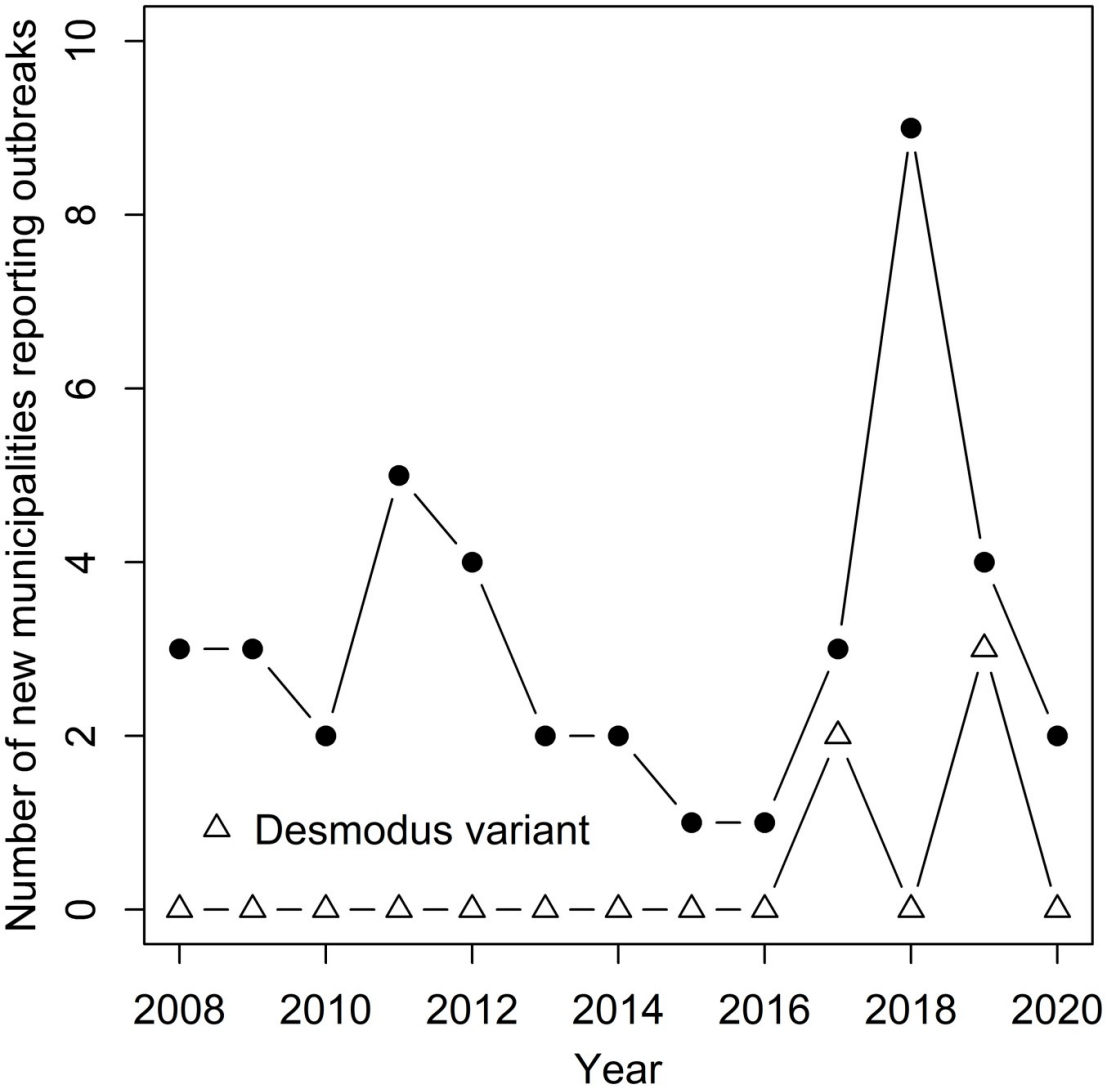
Geographical expansion of rabies reported in *Callithrix jacchus*. Number of municipalities reporting outbreaks for the first time each year from 2008 to October 2020. Open triangles show outbreaks belonging to the *Desmodus rotundus* variant (AgV3).

The total number of municipalities reporting outbreaks was 41, with an average of 5.1 municipalities/year [1–14] (Fig 3). The geographical area reporting outbreaks in *C*. *jacchus* increased though time, with a mean of 3 [1–9] new municipalities reporting outbreaks for the first time each year, which peaked in 2011 with 5 new municipalities and 2018 with 9 new municipalities (Figs 2 and 3). For example, outbreaks were detected for the first time in 2013 in the State of Piauí, in 2017 in Bahia, and in 2019 in Rio de Janeiro (Fig 2). The annual number of new municipalities reporting outbreaks was not significantly correlated to the annual number of dead non-human primates reported to the Ministry of Health at the country level nor the state level (Spearman’s test, p > 0.05), despite a national peak in new municipalities reporting cases in 2018 coinciding with the largest number of dead non-human primates reported across the country (Figs 1 and 3). The year of the first municipality reporting an outbreak of rabies in *C*. *jacchus* since 2008 in the states of Ceará, Pernambuco and Piauí was also the year of the first dead primate submitted for rabies testing to the national surveillance, whereas the states of Bahia and Rio de Janeiro reported dead primates in years prior to their first rabies outbreak in *C*. *jacchus* reported by a municipality (S2 Table).

Several municipalities in different states reported two or more outbreaks of rabies in *C*. *jacchus* in different years including Salvador in Bahia (outbreaks of *D*. *rotundus* variant in 2017 and 2019), Afrânio (unknown variant in 2008, 2010 and 2015) and São José do Belmonte (*C*. *jacchus* variant in 2018 and 2019) in Pernambuco, and Teresina in Piauí (unknown variant in 2018 and *C*. *jacchus* variant 2019). Likewise, thirteen municipalities of Ceará reported two or more outbreaks including Amontada (unknown variant in 2009 and 2013), Aquiraz (outbreaks of unknown variant in 2008 and *C*. *jacchus* variant in 2018), Barroquinha (unknown variant in 2009, 2011, 2012 and 2020), Cascavel (unknown variant in 2008, 2013 and 2015), Crato (unknown variant in 2009 and 2012), Croatá (unknown variant in 2011 and 2020), Eusébio (unknown variant in 2011 and *C*. *jacchus* variant in 2019), Guaraciaba do Norte (unknown variant in 2014 and 2015), Ibiapina (unknown variant in 2013, *C*. *jacchus* variant in 2018 and *D*. *rotundus* variant in 2019), Redenção (unknown variant in 2015 and *C*. *jacchus* variant in 2018), São Benedito (unknown variant in 2011 and 2013), Tianguá (unknown variant in 2011 and 2013) and Uruburetama (unknown variant in 2011 and *C*. *jacchus* variant in 2017 and 2018).

### Occurrence of *C*. *jacchus*

**Fig 4:**
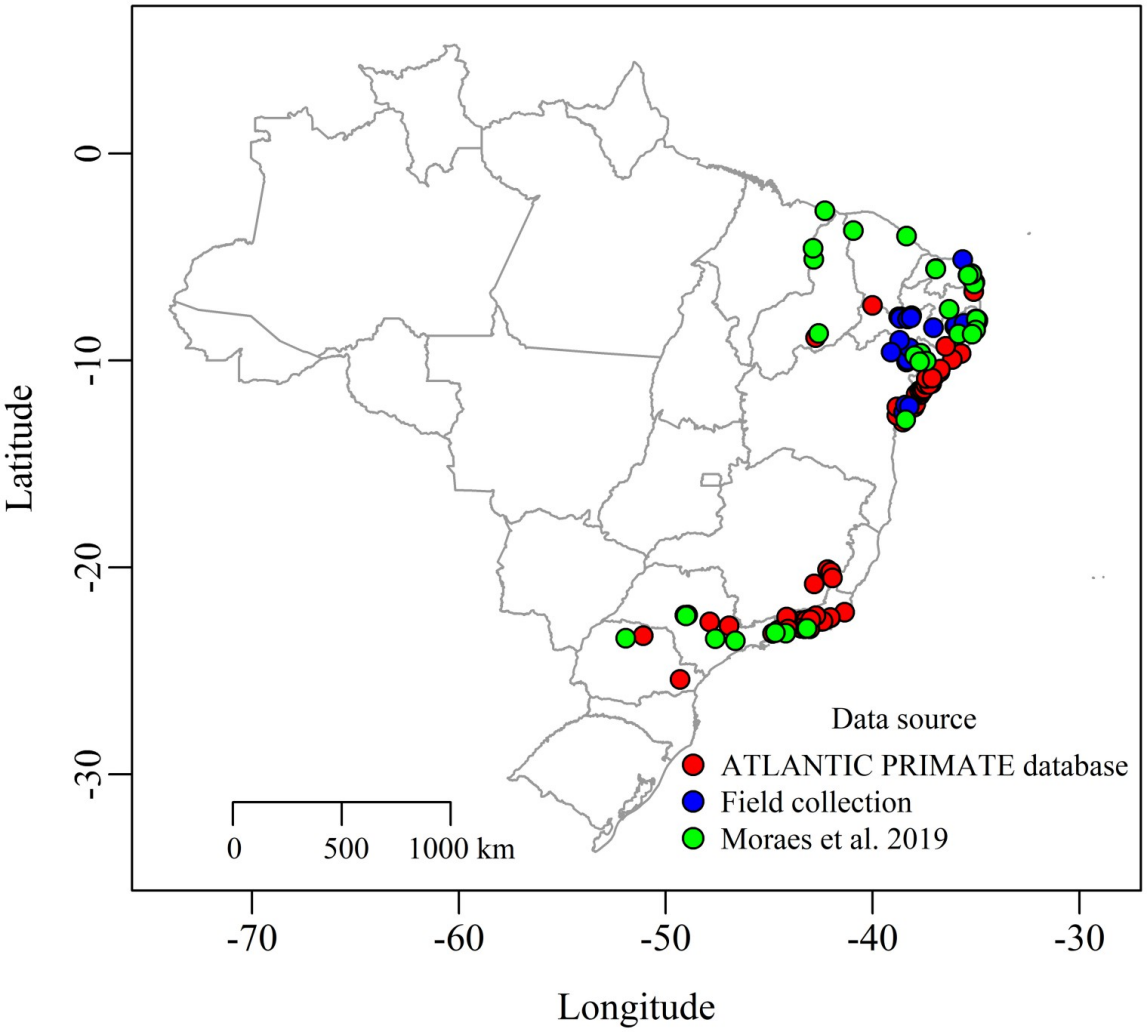
Presence only occurrence locations of *Callithrix jacchus* in Brazil. Red dots show locations published in the ATLANTIC PRIMATES database, blue dots show locations identified during the field missions performed within this study, and green dots show locations extracted from Moraes *et al*. 2019 [23]. Country, region and district maps were obtained from the GADM (http://www.gadm.org//) database, freely-available for academic use, using the *getData* function from the *raster* package of R (map layer can be found here: https://biogeo.ucdavis.edu/data/gadm3.6/Rsp/gadm36_BRA_2_sp.rds)).

The observed distribution of *C*. *jacchus* was divided into 2 distinct regions (1) the northeast of Brazil both in the coast and mainland described as the native range of the species and (2) the south of Brazil including mainly the states of Sao Paulo, Paraná, Rio de Janeiro and Minas Gerais that are considered areas of marmoset introduction (Fig 4). No occurrence was reported more than 600 km east of the coast.

### Drivers of occurrence and predicted habitat suitability for *C*. *jacchus*

The occurrence dataset used for the ecological niche model included 34 duplicate records with the same geographic coordinates, which were removed. A 50 km distance between occurrences resulted in a visually desirable occurrence dataset that was minimally autocorrelated (S1 Text). Therefore, analyses were performed with this set of occurrences. Rarefaction of occurrences randomly removed 87 occurrences, resulting in 57 unique occurrence locations used in our niche modeling, randomly selecting half of locations (n = 29) for model calibrating and the other half (n = 28) for testing.

Five bioclimatic variables were selected by the jackknife procedure as main drivers of the occurrence of *C*. *jacchus* including mean diurnal range (Bioclim 2), temperature annual range (Bioclim 7), the mean temperature of coldest quarter (Bioclim 11), precipitation seasonality (Bioclim 15), and precipitation of driest quarter (Bioclim 17). The jackknife plot of a first global model fitted with *C*. *jacchus* occurrence data and all bioclimatic variables indicated that variables 3 (Isothermality), 16 (Precipitation of the wettest quarter), and 13 (Precipitation of the wettest month) contributed the least to the model. These variables were removed and a subsequent model was refitted with the remaining variables, which further revealed that variables 4 (Temperature seasonality), 5 (Maximum temperature of the warmest month), 6 (Minimum temperature of the coldest month), and 12 (Annual precipitation) were less important to the overall fitness of the model. In a third and final jackknife step, variables 1 (Annual mean temperature), 10 (Mean temperature of the warmest quarter), and 14 (Precipitation of the driest month) were removed and the model evaluated. Table 1 shows variables kept in different jackknife steps and the three environmental variable sets kept for model calibration.

To explore model complexity over the environmental space, we tested the following eight regularization parameter values 0.1, 0.3, 0.5, 0.75, 1, 2, 3, 5, and five feature classes response types including linear, linear and quadratic combined, linear, quadratic and product combined, linear, product, quadratic and threshold combined, and linear, product, quadratic, threshold and hinge combined. With these, the 3 environmental variable sets were simulated. Altogether, this resulted in 120 candidate models. Of these, 98 models were statistically significant, 49 models met the omission criteria (< 10%), 30 statistically significant models met the omission criteria, and one statistically significant model met the omission and the AICc criteria (Details on each model results are given in S4 Table). This final model (AICc = 1506) was built with a regularization multiplier value of 3, linear, product, quadratic, threshold and hinge combined product features, and included all bioclimatic variables from Set 1 (Table 1). The mean AUC ratio of this model was 1.234, omission rate (at 10%) = 0.074, and there were 6 parameters in the model. All interpretations were made from this model alone.

**Fig 5:**
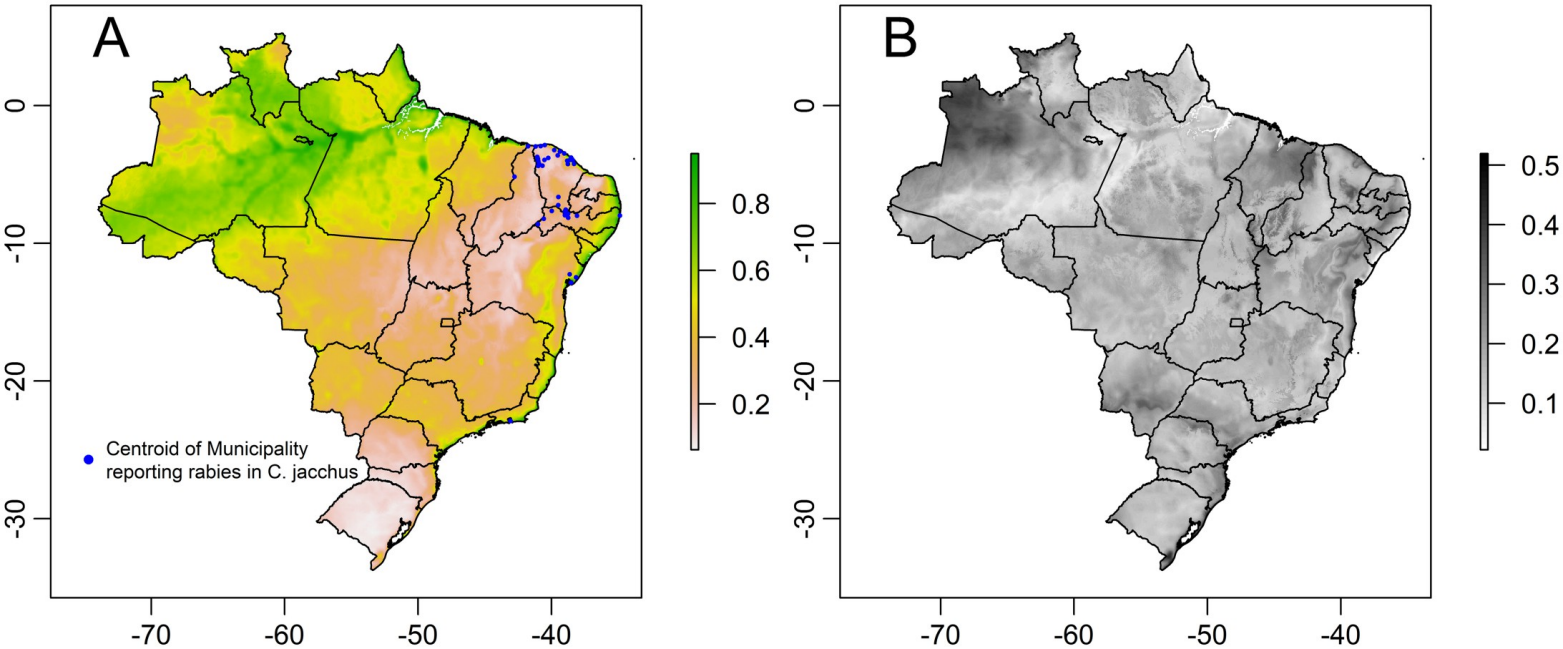
Model predictions on *Callithrix jacchus* suitable area in Brazil. (A) Predicted suitable areas of *Callithrix jacchus* across Brazil (median values of the best fitting model). Areas in darker red indicate higher suitable areas predicted by the best Maxent model. Blue dots represent the centroid of municipalities that have reported a rabies case in *C*. *jacchus* (B) Uncertainty associated with the predicted suitable areas of *Callithrix jacchus* in Brazil. Uncertainty is represented by the range of the model predictions (max-min). Country, region and district maps were obtained from the GADM (http://www.gadm.org//) database, freely-available for academic use, using the *getData* function from the *raster* package of R (map layer can be found here: https://biogeo.ucdavis.edu/data/gadm3.6/Rsp/gadm36_BRA_2_sp.rds).

The predicted habitat suitability of *C*. *jacchus* is given in Fig 5A and model uncertainty in model predictions (range) is given in Fig 5B. The model predicted high habitat suitability for *C*. *jacchus* in most of the coast of Brazil and particularly the east coast (Fig 5A). High habitat suitability was also observed for regions where *C*. *jacchus* has not been reported including north and west of the Amazon. The highest levels of model uncertainty (i.e. range) were observed in the western state of Amazonas and some areas of the northeast such as the state of Maranhão (Fig 5B).

### Predicted habitat suitability of municipalities reporting rabies cases in *C*. *jacchus*

The habitat suitability probability predicted by our Maxent model for the 41 municipalities reporting rabies cases in *C*. *jacchus* ranged from 0.14 to 0.93, with a median, mean ±SD = 0.31,0.40 ±0.21. Municipalities with a poor habitat suitability were mainly located on inland areas of the Caatinga biome (Fig 5A).

## Discussion

Human cases of rabies spillover transmitted by the common marmoset *C*. *jacchus* have emerged in Brazil over the last two decades, and *C*. *jacchus* is now considered the only non-human primate reservoir of rabies [4,6]. Our results show that 67 outbreaks (91 cases) of rabies affecting *C*. *jacchus* have been reported to the Brazilian Ministry of Health between January 2008 and October 2020, with a mean of 5 outbreaks/year ranging from 1 in 2016 to 14 in 2018. Despite no temporal increase in the annual number of outbreaks/municipalities reporting rabies, outbreaks were increasingly reported in new areas, with a mean of three municipalities reporting outbreaks for the first time each year. For example, outbreaks were mainly concentrated in the states of Ceará and Pernambuco up to 2012 but are now also reported in the state of Piauí (since 2013), Bahia (2017), and Rio de Janeiro (2019), with the two latest states only reporting cases due to spillover with the *D*. *rotundus* antigenic variant (AgV3). The Maxent ecological modelling confirmed the high habitat suitability for this species in the northeast and coast of Brazil, but also in the north and west of Brazil. However, the 41 municipalities reporting rabies cases in *C*. *jacchus* varied widely in their habitat suitability probability predicted by our model for *C*. *jacchus*, with poor habitat suitability predicted for several municipalities located on inland areas of the Caatinga biome.

Our analyses show that outbreaks of rabies in *C*. *jacchus* are reported every year in Brazil in northern states, with a fluctuating number of reports. This suggests ongoing circulation of the virus on *C*. *jacchus* populations, with unknown consequences for its populations and public health. The northern region of the Ceará state and the eastern region of the Pernambuco state bordering Ceará reported most outbreaks (Fig 4). Seventeen municipalities (13 from Ceará) reported two or more outbreaks in different years, representing potential hotspots for rabies transmission. The municipality of Ibiapina even reported the *C*. *jacchus* (2018) and *D*. *rotundus* (2019) variants, suggesting the circulation of two independent epidemiological rabies cycles in the same area. Yearly fluctuations in the number of reported cases could be due to both annual changes in the circulation of rabies among *C*. *jacchus* or the detection capacity of dead animals that are tested for rabies. Although no significant correlation was observed between annual rabies cases and the total annual number of dead non-human primates reported to the Ministry of Health at the country level, there was a significant correlation in the state of Ceará, which reports most of the rabies cases in *C*. *jacchus*. Furthermore, the peak of outbreaks reported in 2018 coincide with an increased in passive surveillance of dead primates submitted and tested for rabies in Brazil due to a major outbreak of Yellow Fever affecting non-human primates and humans in southern states between 2016 and 2018, although no peak in rabies outbreaks was observed in 2017 when surveillance was also at its highest [37]. Therefore, our results suggest that high numbers of yearly outbreaks of rabies in *C*. *jacchus* reported could be at least partially due to an increase of passive surveillance and that the full extent of the circulation of rabies among *C*. *jacchus* is likely underestimated.

Our spatiotemporal analyses showed an increase in the total cumulative number of municipalities reporting rabies in *C*. *jacchus* with time, without a significant correlation with the total number of dead non-human primates submitted at the state or national level, except for a similar peak in 2018. Most outbreaks in Pernambuco of the *C*. *jacchus* variant emerged after 2017. Spatial expansions of rabies in wild reservoirs such as vampire bats are common [38]. Our study suggests that spatial expansions could be also occurring in rabies transmitted by *C*. *jacchus*, requiring further research on the mechanisms driving these expansions. Alternatively, the increase of new municipalities and states reporting rabies in *C*. *jacchus* that could be interpreted as rabies spatial expansions in could also be explained by increased surveillance capacity in these areas over time. Further studies should therefore discriminate between these two alternatives by estimating and comparing *C*. *jacchus* rabies surveillance capacity in municipalities with and without rabies reported.

Despite ongoing transmission and a bite incidence of around 2.0 primate bites /100,000 habitants/year on states reporting rabies in *C*. *jacchus* [9], 19 human cases of rabies transmitted by *C*. *jacchus* were reported between 1990 and 2016 [4,6]. This relatively limited number of cases reported in humans suggests either a low prevalence of rabies in *C*. *jacchus*, low transmission of rabies from *C*. *jacchus* to humans when outbreaks of rabies in *C*. *jacchus* occur, effective post-exposure prophylaxis (PEP) of bitten patients, or potential under-reporting of human cases associated with rabies transmission from *C*. *jacchus*. A recent study evaluating the efficiency of PEP administration in Brazil have shown that only half of patients bitten by primates attending local public health centers received the appropriate PEP recommended by the Ministry of Health [9], which favors the other hypotheses to explain the low number of cases reported among humans.

The current spatial distribution of the common marmoset is only anecdotally known throughout the aggregation of published records on its occurrence [18] and the area in which it could successfully established in Brazil after human-mediated invasions remains poorly understood. Our Maxent model predicts that the northeast coast of Brazil including the Atlantic forest and Caatinga biomes is suitable habitat for this species, similar to other recent modelling results and previous knowledge of *C*. *jacchus* been endemic to the northeast of Brazil [22,23]. In agreement with the study of Moraes *et al*. (2019), our model predicts that coastal regions from the northern state of Ceará to the southern state of Santa Catarina are suited habitat for the establishment of *C*. *jacchus* if it invades these areas in the coming years, which has already occurred in the states of Sao Paulo and Rio de Janeiro. Our model also predicts other new inland areas that are highly suitable for *C*. *jacchus* in the west of the country in regions bordering Bolivia and Peru (e.g. states of Acre, Rondonia and Amazonas) as well as in the north of the country (e.g. states of Roraima, Amapá, Pará and Amazonas). Previous studies have shown that the distribution of *C*. *jacchus* depends more on climate variables than biotic factors such as competition with other species of marmosets [22] and further expansions of *C*. *jacchus* range are expected as a result of climate change [22]. Considering the current pet trade practices of *C*. *jacchus* that have resulted in the invasion of this species in the south of Brazil, our model predictions suggest that the probability of establishment of *C*. *jacchus* is significant in the Amazonian region if the species was transported to that area. Thus, appropriate steps to prevent the movement and introductions of *C*. *jacchus* into those areas should be considered, since the rabies outbreak of *C*. *jacchus* in the state of Rio de Janeiro confirms the potential for rabies spillover from *D*. *rotundus* associated with invasion of *C*. *jacchus* [13].

In the present study, we used Maxent ecological niche modelling to predict the entire niche suitability of the *C*. *jacchus* in Brazil that included recent methodological improvements in Maxent modelling. Several ecological niche modeling approaches can be used to obtain predicted habitat suitability maps for a given species [39] and have been applied in conservation, invasive species ecology, and disease related systems such as vector distribution (e.g. [40–44]). Ecological niche modeling using Maxent is a well-tested approach for estimating species distributions based on abiotic factors. Despite its utility, some pitfalls in niche modeling have been identified, and new recommendations have been made for building robust models including the appropriate thinning of occurrence data [45], consideration of an accessible area (M) for the studied species [26], model calibration to explore model complexity [35,45], and the use of more than one statistical criterion for model selection [46–48]. All these recommendations were considered in this study to produce a robust spatial distribution model for *C*. *jacchus*. This, along with the model’s very low-omission rate (0.07%) gives high confidence of the predicted suitability area for this species in Brazil given the available occurrence data, and that the environmental variables used in the modeling were adequate.

Despite applying several improvements in our Maxent modelling and using a rarefication procedure to avoid biasing our model predictions to areas with high sampling effort, most areas of the semi-arid Caatinga biome in the northeast of Brazil such as inland regions of the states of Pernambuco and Ceará, considered as natural habitat for *C*. *jacchus*, were predicted as low habitat suitability for *C*. *jacchus* by our model and previous models [23]. Therefore, future research could contribute to improve our model predictions on *C*. *jacchus* occurrence, ecology, and rabies risk. For example, the low habitat suitability predicted by our model for *C*. *jacchus* populations in the Caatinga Biome could reflect poor sampling effort in these inland areas affecting model predictions, a relative lower density of *C*. *jacchus* compared to coastal areas, or adaptation of *C*. *jacchus* to an environment predicted as ‘poor quality’ by the bioclimatic variables used in current models [49]. In fact, *C*. *jacchus* living in the challenging conditions of the Caatinga biome display several differences in behavior, diet, group size and home range compared to populations living in the Atlantic Forest [49–50]. These adjustments in behavior and diet could explain their presence in areas predicted as low habitat suitability based only in ecological variables. Furthermore, municipalities reporting rabies cases had a habitat suitable probability ranging from low to high, with an averaged low probability on inland areas of the Caatinga biome (Fig 5A). Therefore, accurately predicting rabies risk based on *C*. *jacchus* presence would require understanding if areas of low habitat suitability predicted in our model where *C*. *jacchus* is present are particularly suited for rabies transmission. For example, behavioral adjustments of *C*. *jacchus* in the Caatinga biome (e.g. larger home range [50]) could impact the dynamics of rabies transmission dynamics in populations of this biome.

Rabies virus circulates in a specific viral strain among the common marmoset within its endemic range [4]. However, recent reports of rabid *C*. *jacchus* infected by the common vampire bat *D*. *rotundus* variant of the virus (AgV3) in Rio de Janeiro [13], Bahia and Ceará are emerging. These reports suggest that *C*. *jacchus* could also act as an incidental host (e.g. sentinel) reflecting transmission of rabies among vampire bats within its natural range and in areas of *C*. *jacchus* invasion. Since variant detection started only in 2017 with the first outbreak of *D*. *rotundus* variant detected in *C*. *jacchus*, the existence of previous spillover events of rabies from *D*. *rotundus* to *C*. *jacchus* is unknown. The expansion of rabies risk to humans by either the *C*. *jacchus* variant or spillover from *D*. *rotundus* will depend on the possibility of transporting infected individuals into these new areas or creating new interactions between *C*. *jacchus* and bats. The transportation of infected individuals can be limited by reducing both the legal and illegal pet trade and laboratory use (and subsequent release into natural areas) of this species across Brazil. Likewise, reducing contact between infected marmosets and humans will depend on efficient awareness campaigns explaining to the population at risk, for example, how to identify rabid animals. These campaigns could rely on current participative initiatives to survey Yellow Fever symptoms in primates across the country, but should be cautious not to stigmatize *C*. *jacchus* as this could have unintended and negative consequences such as promoting primate killings as observed for howler monkeys during Yellow Fever epidemics [51].

The potential expansion of rabies in *C*. *jacchus* to new areas calls for serological studies to identify areas of *C*. *jacchus* where rabies is circulating, and to estimate whether the low number of primate deaths submitted for rabies testing in states where rabies is circulating (e.g. state of Piauí) reflects low rabies circulation among *C*. *jacchus* or low surveillance capacity. Future population genetic studies can also contribute to elucidate if *C*. *jacchus* is expanding its natural distribution in the northeastern Brazil and to identify the human-induced routes generating *C*. *jacchus* invasions to the south, as well as if hybridization with other marmoset species (e.g. *C*. *penicillata* in the south [23]) could affect their susceptibility to rabies. Overall, the highly invasive characteristics of *C*. *jacchus* calls for the implementation of targeted measures to prevent this rabies reservoir from further invasions in Brazil, which could increase the risk of rabies human spillover.

## Supporting information

S1 Table Rabies cases reported for *C*. *jacchus*. (CSV)

S2 Table *C*. *jacchus* GPS locations used in this study. (XLSX)

S3 Table Annual number of dead non-human primates reported for rabies testing by each state. (CSV)

S4 Table ENM model candidates and parameter estimations. (CSV)

S1 Text Spatially rarefied occurrence data for ENM modelling. The Brazil map was obtained from the GADM (http://www.gadm.org//) database, freely-available for academic use under CC BY license, using the *getData* function from the *raster* package of R (map layer can be found here: https://biogeo.ucdavis.edu/data/gadm3.6/Rsp/gadm36_BRA_2_sp.rds)).
(DOCX)
