# Supplementary material for: Spatio-temporal dynamics of rabies and habitat suitability of the common marmoset Callithrix jacchus in Brazil
Source: PLoS Negl Trop Dis. 2022 Mar 31;16(3):e0010254. doi: 10.1371/journal.pntd.0010254 (PMC8970506; doi:10.1371/journal.pntd.0010254)
Supplement: S1 Text — The Brazil map was obtained from the GADM (http://www.gadm.org//) database, freely-available for academic use under CC BY license, using the getData function from the raster package of R (map layer can be found here: https://biogeo.ucdavis.edu/data/gadm3.6/Rsp/gadm36_BRA_2_sp.rds)). (DOCX) [file pntd.0010254.s005.docx]

Occurrence data rarified using the SDM toolbox for the MaxEnt ecological niche model

**Rarefy with a 10 km radius between occurrence points** Initial points: 178

Duplicates: 34 removed

Spatially autocorrelated: 38

Final dataset: 106 unique occurrence points

Map of Brazil showing the selected points:

The Brazil map was obtained from the GADM ([http://www.gadm.org//](http://www.gadm.org/)) database, freely-available for academic use under CC BY license, using the getData function from the raster package of R (map layer can be found here: <https://biogeo.ucdavis.edu/data/gadm3.6/Rsp/gadm36_BRA_2_sp.rds)>.


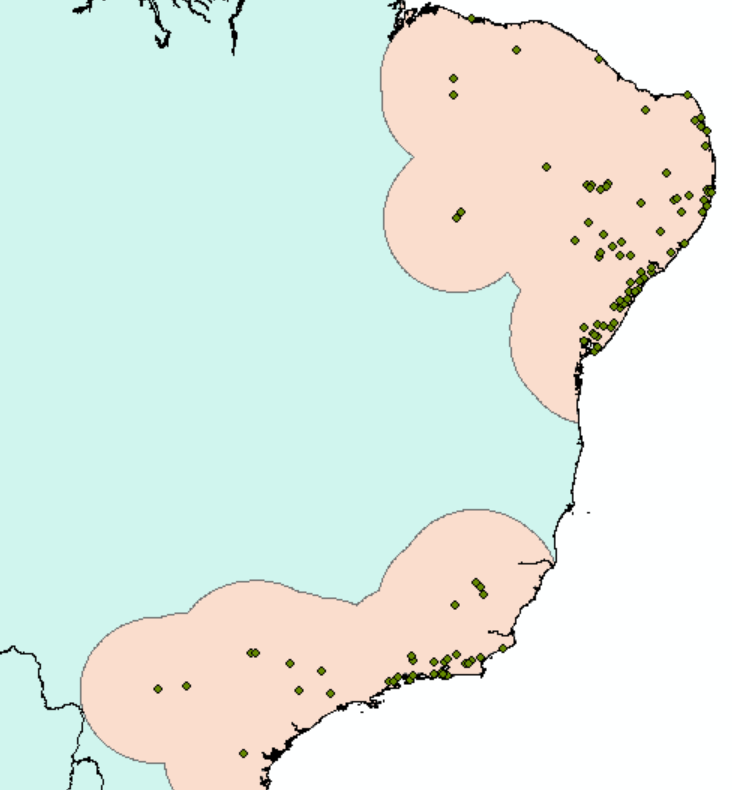


**Rarefy with a 25 km radius between occurrence points**

Initial points: 178

Duplicates: 34 removed

Spatially autocorrelated: 63

Final dataset: 81 unique occurrence points

Map of Brazil showing the selected points:

The Brazil map was obtained from the GADM ([http://www.gadm.org//](http://www.gadm.org/)) database, freely-available for academic use under CC BY license, using the getData function from the raster package of R (map layer can be found here: <https://biogeo.ucdavis.edu/data/gadm3.6/Rsp/gadm36_BRA_2_sp.rds)>.


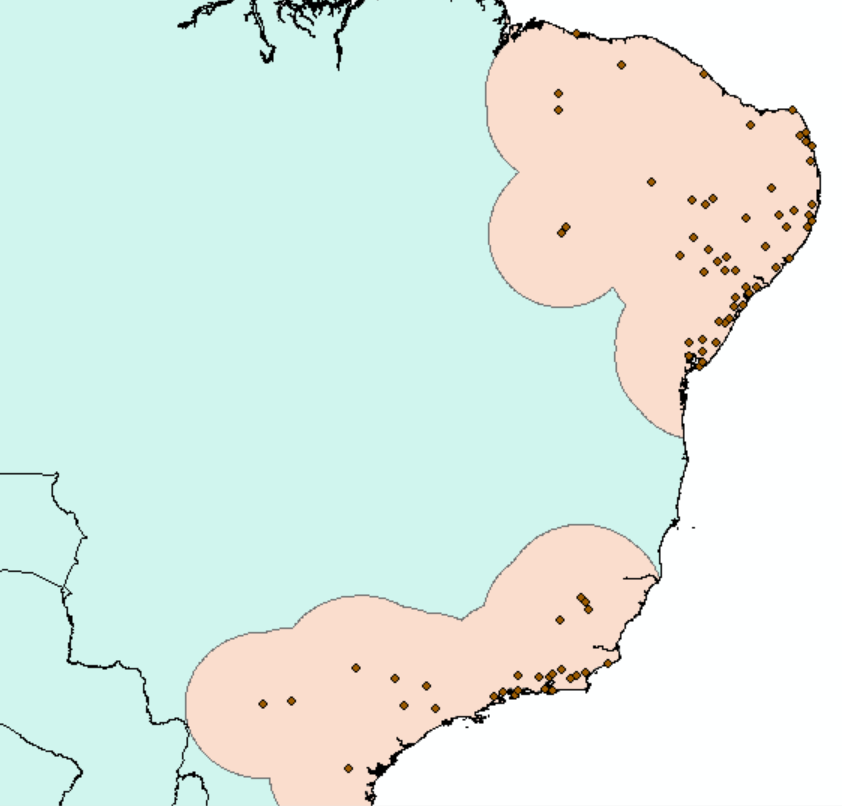


**Rarefy with a 50 km radius between occurrence points**

Initial points: 178

Duplicates: 34 removed

Spatially autocorrelated: 87

Final dataset: 57 unique occurrence points

Map of Brazil showing the selected points:

The Brazil map was obtained from the GADM ([http://www.gadm.org//](http://www.gadm.org/)) database, freely-available for academic use under CC BY license, using the getData function from the raster package of R (map layer can be found here: <https://biogeo.ucdavis.edu/data/gadm3.6/Rsp/gadm36_BRA_2_sp.rds)>.


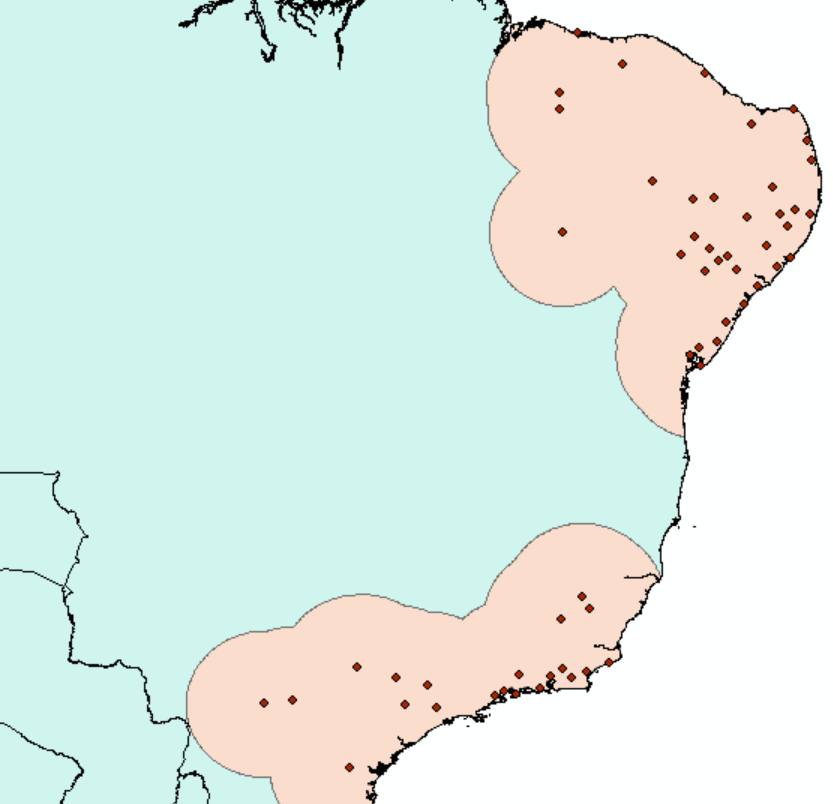


50 km distance between occurrences resulted in a visually desirable occurrence dataset that is minimally autocorrelated. Therefore, analysis were performed with this set of occurrences.
